# Comparing two extracellular additives to facilitate extended storage of red blood cells in a supercooled state

**DOI:** 10.3389/fphys.2023.1165330

**Published:** 2023-06-01

**Authors:** Nishaka William, Ziya Isiksacan, Olga Mykhailova, Carly Olafson, Martin L. Yarmush, O. Berk Usta, Jason P. Acker

**Affiliations:** ^1^ Department of Laboratory Medicine and Pathology, University of Alberta, Edmonton, AB, Canada; ^2^ Center for Engineering in Medicine and Surgery, Massachusetts General Hospital, Harvard Medical School, Boston, MA, United States; ^3^ Shriners Children’s, Boston, MA, United States; ^4^ Innovation and Portfolio Management, Canadian Blood Services, Edmonton, AB, Canada; ^5^ Department of Biomedical Engineering, Rutgers University, Piscataway, NJ, United States

**Keywords:** supercooling, storage lesion, red blood cells, trehalose, polyethylene glycol, surface-sealing, nucleation

## Abstract

**Background:**

Adenosine triphosphate (ATP) levels guide many aspects of the red blood cell (RBC) hypothermic storage lesions. As a result, efforts to improve the quality of hypothermic-stored red cell concentrates (RCCs) have largely centered around designing storage solutions to promote ATP retention. Considering reduced temperatures alone would diminish metabolism, and thereby enhance ATP retention, we evaluated: (a) whether the quality of stored blood is improved at −4°C relative to conventional 4°C storage, and (b) whether the addition of trehalose and PEG400 can enhance these improvements.

**Study Design and Methods:**

Ten CPD/SAGM leukoreduced RCCs were pooled, split, and resuspended in a next-generation storage solution (i.e., PAG3M) supplemented with 0–165 mM of trehalose or 0–165 mM of PEG400. In a separate subset of samples, mannitol was removed at equimolar concentrations to achieve a fixed osmolarity between the additive and non-additive groups. All samples were stored at both 4°C and −4°C under a layer of paraffin oil to prevent ice formation.

**Results:**

PEG400 reduced hemolysis and increased deformability in −4°C-stored samples when used at a concentration of 110 mM. Reduced temperatures did indeed enhance ATP retention; however, in the absence of an additive, the characteristic storage-dependent decline in deformability and increase in hemolysis was exacerbated. The addition of trehalose enhanced this decline in deformability and hemolysis at −4°C; although, this was marginally alleviated by the osmolarity-adjustments. In contrast, outcomes with PEG400 were worsened by these osmolarity adjustments, but at no concentration, in the absence of these adjustments, was damage greater than the control.

**Discussion:**

Supercooled temperatures can allow for improved ATP retention; however, this does not translate into improved storage success. Additional work is necessary to further elucidate the mechanism of injury that progresses at these temperatures such that storage solutions can be designed which allow RBCs to benefit from this diminished rate of metabolic deterioration. The present study suggests that PEG400 could be an ideal component in these solutions.

## Introduction

Red blood cell (RBC) storage is a critical component of transfusion infrastructures that globally allow 108 million individuals each year to receive red cell concentrates (RCCs) as a therapeutic strategy to curb tissue oxygenation deficits (Carson et al., 2012). Hypothermic preservation at 4°C and cryopreservation at −80°C or −196°C, respectively, allow for the short- and long-term storage of RCCs (Scott et al., 2005). However, both are subject to limitations that in turn warrant concurrent efforts to facilitate both their refinement as well as the development of alternative storage strategies. While there are encouraging results poised to marginally improve the standards of current RCC preservation strategies, efforts to develop alternatives to these current standards have been nominal (Yoshida and Shevkoplyas, 2010; Capicciotti et al., 2015; Deller et al., 2015; Lagerberg et al., 2017; Dou et al., 2019; Zhu et al., 2019).

Hypothermic preservation for ∼42 days is considered the state-of-the-art, as RCC cryopreservation is generally reserved for rare blood types due to concerns surrounding its ability to meet heightened or immediate blood demands (*e.g.*, during military/natural disasters requiring high numbers of massive transfusions). Although both storage methods have seen advances in recent decades (Deller et al., 2015; Dou et al., 2019; Zhu et al., 2019; D'Alessandro et al., 2015b; D'Alessandro et al., 2018; Lagerberg et al., 2017), the regulatory approval and implementation of said developments have been significantly more prominent for RCC cryopreservation (Scott et al., 2005). This is in part attributed to the long-held perception that currently used storage solutions and methods for the hypothermic storage of RCCs are satisfactory and do not necessitate appreciable improvements (Högman et al., 1983). However, in 2008, a retrospective study published in the *New England Journal of Medicine* highlighted an increase in the morbidity and mortality of cardiac surgery patients transfused with units that exceeded 14 days of storage (Koch et al., 2008). While these findings were highly controversial, they have since been corroborated by several other retrospective studies as well as some preclinical studies which suggest that “older” stored RCCs (>14 or 21 days) could promote endothelial dysfunction and pathophysiologic consequences in certain patient populations (Baek et al., 2012; Kanias and Gladwin, 2012; Pavenski et al., 2012; Risbano et al., 2015). These findings have urged many leaders in the field to reconsider the limitations of hypothermic storage.

Efforts to improve the hypothermic storage of RCCs have largely centered around the redesign of storage solutions such that the depletion of adenosine triphosphate (ATP) is alleviated (D'Alessandro et al., 2015a; Lagerberg et al., 2017; D'Alessandro et al., 2015b; D'Alessandro et al., 2018; De Korte et al., 2008). ATP reservoirs begin to drop after 2–3 weeks of storage and reside at approximately 40% of their original value at expiry, driving many of the storage-induced changes in RBC rigidity and morphology that impede *in vivo* function (D'Alessandro et al., 2015a). While slowed at hypothermic temperatures, RBC metabolism does proceed, resulting in continuous depletion of ATP. This is a consequence of continued lactic acid generation that reduces the intracellular pH and promotes subsequent inhibition of glycolytic activity. Many of the more prominent next-generation storage solutions (*i.e.*, SOLX, PAGGSM, PAG3M) are designed to favour intracellular alkalosis by having a higher starting pH and/or promoting chloride efflux (*i.e.*, exploiting the chloride-shift concept) through a high bicarbonate load, a low-chloride concentration, or an absence of chloride (Lagerberg et al., 2017). To further minimize ATP depletion, PAGGSM and PAG3M additionally contain guanosine as a source of ribose phosphate to drive additional late-stage glycolytic ATP production without requiring any additional net ATP expenditure (D'Alessandro et al., 2018). Of the next-generation storage solutions developed thus far, PAG3M has been the most effective with respect to its ability to minimize intracellular acidification and in turn maintain high levels of ATP throughout storage (∼50% higher levels of ATP at expiry relative to the conventional storage solutions) (Lagerberg et al., 2017).

ATP levels guide key aspects of the hypothermic storage lesions and are the most potent metabolic correlates of RBC post-transfusion survival (Luten et al., 2008). Thus, we posit that lowering the storage temperature would further diminish metabolic deterioration, improve the quality of stored RCCs, and perhaps even lengthen the allowable storage duration (De Korte et al., 2008). Preservation in an unfrozen state at high sub-zero temperatures (i.e., supercooling) down to −15°C has been a recently popularized strategy to avoid ice-induced damage and extend organ storage limits that currently contribute to organ shortages (Monzen et al., 2005; Berendsen et al., 2014; de Vries et al., 2019). While supercooling has been considered as a strategy to store a variety of different cell types of therapeutic interest, supercooled storage of RBCs has only recently received attention (Usta et al., 2013; Puts et al., 2015; Huang et al., 2018; Huang et al., 2020; Zhu et al., 2022). One of the major challenges with supercooling is to ensure that ice formation does not occur in the metastable “supercooled” state that exists between the liquid-solid phase transition; an objective that is exceedingly hard to achieve in large-volume systems like RCCs. However, we have recently published on the ability of immiscible hydrocarbon solutions to lower the probability of ice formation at the air-liquid interfaces (the most likely site for ice formation) and facilitate the stable supercooled storage of 100 mL aqueous solutions for 100 days (Huang et al., 2018).

In the present study, we aim to characterize differences in the *in vitro* quality of RBCs stored at −4°C relative to RBCs stored at 4°C in the next-generation PAG3M storage solution as part of our ongoing efforts to establish the feasibility of supercooling as an RBC storage strategy. There are various injuries that could be induced at sub-zero temperatures, with membrane destabilization being one of the most prominent. Thus, we set out to assess whether trehalose or polyethylene glycol, two commonly used compounds known to provide low-temperature stabilization, could improve supercooled RBC preservation (Mack et al., 1991; Xie and Timasheff, 1997; Moussa et al., 2008; Puts et al., 2015; Zhao et al., 2018).

## Materials and methods

### Blood processing and preparation for supercooled storage

Ten ABO/RhD-compatible CPD/SAGM leukoreduced RCCs provided by Canadian Blood Services (CBS) Blood for Research Facility (Centre for Innovation, Vancouver, British Columbia, Canada) were pooled into a 3-L pooling bag (VSE8014XA, Macopharma) using a sterile connecting device (CompoDock, Fresenius Kabi) and an octopus tubing system (Sanquin Blood Bank) within 7 days of blood collection. Pooled RCCs were then split at volume of 20 mL into 50 mL conical tubes and centrifuged at 1,000 *g* (acc. 3, dec. 9) to pellet the RBCs and remove the SAGM. This protocol was approved by the CBS ethics board (2021.013), and all donors provided informed consent.

PAG3M was prepared in-house as previously described and supplemented with either 27.5 mM, 55 mM, 110 mM, or 165 mM of either trehalose (BP2687-25, Fisher Scientific, Waltham, MA, United States of America) or PEG400 (PX1286B-2, Sigma-Aldrich, Oakville, ON, Canada) and added to the packed RBCs at a volume of 10 mL (Lagerberg et al., 2017). To further investigate how the osmolarity of non-permeant solutes could influence the protection imparted by these additives, we removed mannitol at equimolar concentrations to achieve a fixed osmolarity between the additive and non-additive groups. These osmolarity adjustments were solely done for the 27.5 mM and 55 mM additive concentrations as the concentration of mannitol in PAG3M is 55 mM.

After the RBCs were suspended in their respective solutions (Hct: 50%–60%) two additional wash steps (1,000 x g, acc. 3, dec. 9) were carried out to ensure that all residual SAGM was removed. RBCs from each condition were then added to 5 mL polystyrene tubes (14-959-5, Fisher Scientific) at a volume of 1.5 mL. Samples were then layered with 0.5 mL of paraffin oil (PX0046-1, Sigma-Aldrich) to prevent ice formation and subsequently stored at 4°C ± 1°C and −4°C ± 1°C.

### RBC *in vitro* quality assays

Hemolysis was tested in all samples at day 0 to confirm that the washing in each of the different storage solutions did not accentuate damage (Supplementary Table S1). In un-supplemented samples (*i.e.*, those stored in PAG3M without trehalose or PEG400), hemolysis, deformability, RBC indices, and ATP were evaluated on days 7, 21, 42, and 126 of storage to assess the progression of the storage lesions. For samples supplemented with trehalose or PEG400, each of these quality parameters, apart from ATP, were assessed on day 42. ATP levels were not assessed as we did not suspect these extracellular additives would have an appreciable impact on metabolism. Detailed descriptions on the methods used to assess these *in vitro* quality parameters can be found in previous publications by our group (Almizraq et al., 2013; Acker et al., 2014). In brief, RBC deformability and hematological indices (mean cell hemoglobin (MCH), mean cell volume (MCV), and mean cell hemoglobin concentration (MCHC)) were, respectively, assessed using a laser-assisted optical rotational cell analyzer (Mechatronics, Zwaag, Netherlands) and an automated cell counter (Beckman Coulter, New York, NY). Hemolysis was optically evaluated with a Drabkin’s-based method to determine the ratio of supernatant to total hemoglobin using hematocrit level to account for the volume of supernatant in the sample (Drabkin, 1949). ATP was assessed using a commercially available kit (DiaSys Diagnostic Systems GmbH, Holzheim, Germany) where the RBC samples were mixed in cold 10% trichloroacetic acid to facilitate nucleotide extraction, and the supernatants were then combined with substrates (glucose and NAD+) and enzymes (hexokinase and glucose-6-phosphate dehydrogenase) such that the ATP in the sample could fuel NADH production. The amount of NADH produced was proportional to the amount of ATP, and this was measured spectrophotometrically (SpectraMax 364, Molecular Devices, San Jose, CA, United States).

For assessment of deformability and RBC indices, the samples were washed three times prior to testing (1,000 x g, acc. 9, dec. 3) and resuspended in un-supplemented PAG3M to ensure that the presence of trehalose or PEG400 was not impacting readout of these parameters. This was not done for hemolysis measurements as this would have caused removal of supernatant hemoglobin and limited measurement accuracy.

### Statistical analyses

Statistical analyses were performed using GraphPad Prism 8.1.0. For all analyses, a two-way analysis of variance (ANOVA) followed by a Sidak’s *post hoc* test was performed to evaluate significant differences between conditions (*p* < 0.05).

## Results

**FIGURE 1:**
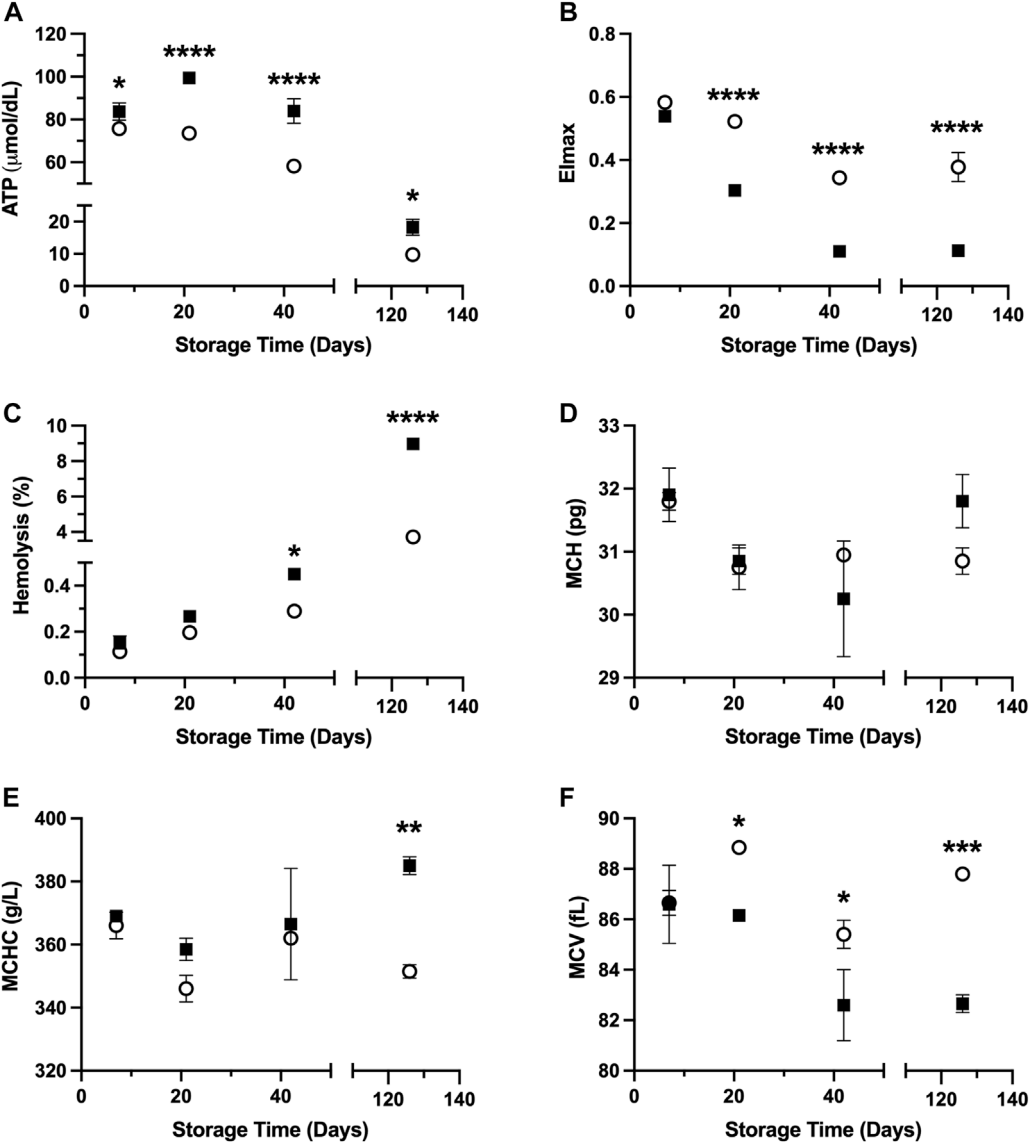
*In vitro* RBC quality parameters following 126 days of storage in the storage solution PAG3M at 4°C (○) and −4°C (■). **(A)** ATP. **(B)** EI_max_. **(C)** Hemolysis. **(D)** MCH. **(E)** MCHC. **(F)** MCV. Error bars for **A**–**C** represent three samples per condition while error bars for **D–F** represent two samples per condition. Error bars are not present in cases where they are smaller than the size of the symbol. Significant differences were calculated using a two-way analysis of variance (ANOVA) followed by a Sidak’s *post hoc* test: *p < 0.05; **p < 0.01; ***p < 0.001; ****p < 0.0001.

Results shown in Figure 1 demonstrate *in vitro* quality parameters for RBCs suspended in PAG3M and stored at hypothermic (4°C) and supercooled (−4°C) conditions. Starting at day 7, ATP retention is improved at −4°C (day 7: *p* = 0.0348; day 21: *p* < 0.0001; day 42: *p* < 0.0001; day 126: *p* = 0.0226) (Figure 1A). Despite this promising indication for supercooled storage, there was a loss in deformability in RBCs stored at −4°C, which started at day 21 (*p* < 0.0001) and continued to day 126 (*p* < 0.0001) (Figure 1B). In agreement with this, hemolysis following storage at −4°C was significantly increased by day 42 (day 42: *p* = 0.046; day 126: *p* < 0.0001) (Figure 1C). With respect to changes in the RBC indices, there was no significant differences in MCH (i.e., the average RBC hemoglobin concentration) between the −4°C- and 4°C-stored conditions at any of the tested timepoints (Figure 1D). MCHC (i.e., the average hemoglobin concentration in a given volume of RBC), however, did prove to be significantly higher at day 126 in the −4°C-stored condition (*p* = 0.0049) (Figure 1E). Furthermore, the MCV (i.e., the average RBC volume) was significantly lower in this condition starting at day 21 (day 21: *p* = 0.0398; day 42: *p* = 0.0332; day 126: *p* = 0.0008) (Figure 1F). Due to the small sample size of these RBC index assessments (i.e., n = 2), however, one must exert caution when interpreting these differences.

**FIGURE 2:**
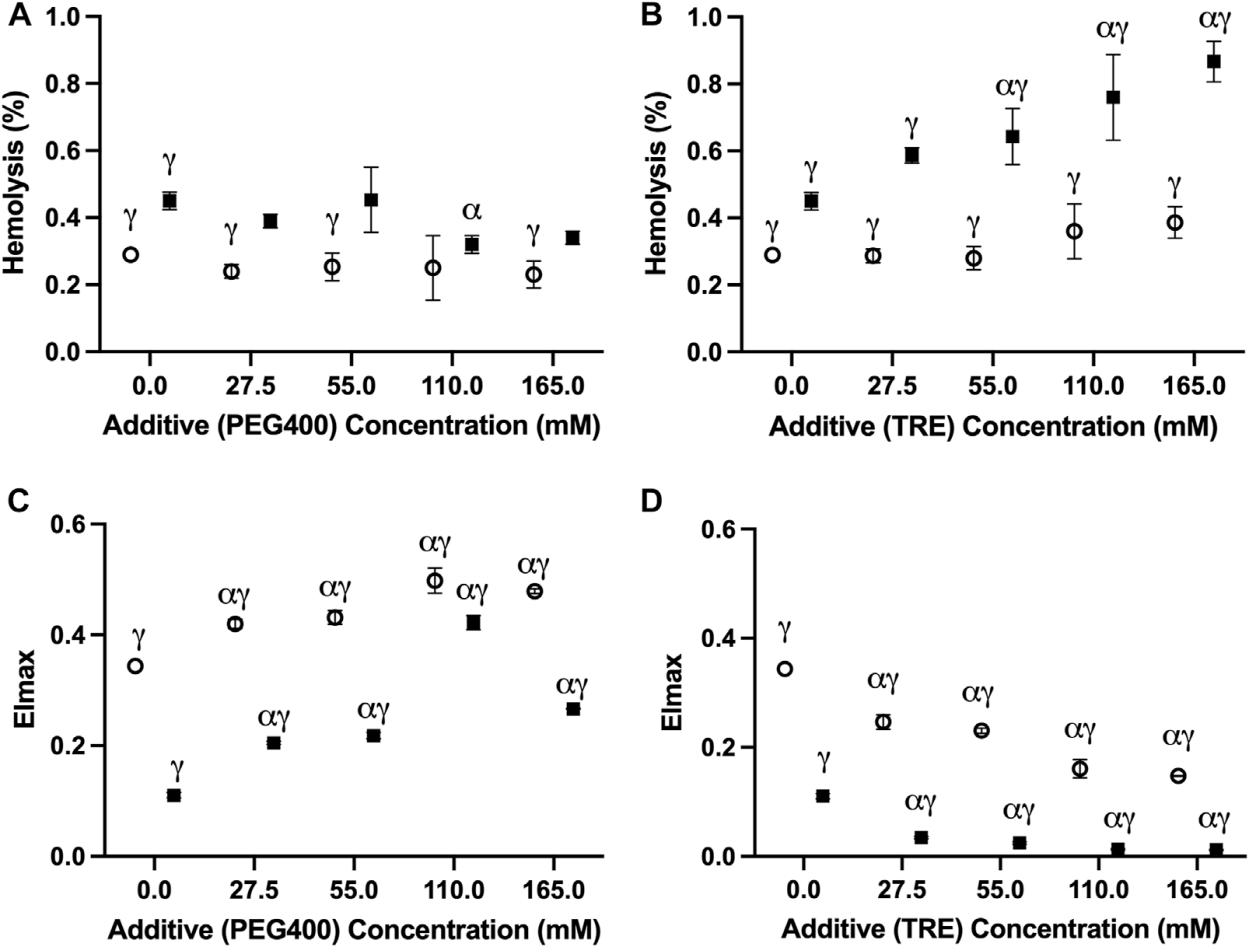
The impact of PEG400 and trehalose on the hemolysis and deformability of RBCs stored for 42 days at 4°C (○) and −4°C (■). **(A)** Hemolysis of PEG400-stored RBCs. **(B)** EI_max_ of PEG400-stored RBCs. **(C)** Hemolysis of trehalose-stored RBCs. **(D)** EI_max_ of trehalose-stored RBCs. All error bars represent the standard deviation of three samples per condition. Error bars are not present in cases where they are smaller than the size of the symbol. Significant differences were calculated using a two-way analysis of variance (ANOVA) followed by a Sidak’s *post hoc* test: α—significant difference relative to the un-supplemented (*i,e.*, 0 mM) condition at the equivalent temperature (*p* < 0.05); γ—significant difference relative to the un-supplemented (*i.e.*, 0 mM) condition at the opposing temperature (*p* < 0.05).

In no case did the addition of PEG400 promote hemolysis at the onset of storage; thus, the changes observed at day 42 of storage are unlikely to be a by-product of potential damage that might have occurred during the initial washing/addition steps (Supplementary Table S1). Of the tested PEG400 concentrations, 110 mM PEG400 offered a significant (*p* = 0.0179), albeit slight (0.13% ± 0.052%) reduction in hemolysis relative to the unsupplemented (i.e., 0 mM PEG400) condition at −4°C; whereas, in no case was a reduction in hemolysis apparent at 4°C (Figure 2A). Interestingly, each of the concentrations (27.5 mM–165 mM) significantly increased EI_max_ (the measure of RBC deformability) in samples stored at −4°C and 4°C (Figure 2B). However, in agreement with the hemolysis results at −4°C, EI_max_ for the 110 mM PEG400 condition was significantly higher than that which was observed at the other concentrations (Figure 2B; *p* < 0.0001 in all cases). Concentrations of 27.5 mM–110 mM and 55 mM–165 mM, respectively, caused a significant increase in MCV at −4°C and 4°C; however, no other differences in other hematological indices were apparent (Table 1).

**TABLE 1:** Hematological indices of RBCs stored for 42 days at 4°C and −4°C^a^.

			MCV (fL)		MCH (pg)		MCHC (g/L)	
			4°C	−4°C	4°C	−4°C	4°C	−4°C
No additive			85.4 ± 4 0.566^b^	83.6 ± 0.00^b^	30.95 ± 0.071	30.25 ± 0.919	362 ± 1.414	361.5 ± 2.121
Trehalose	27.5 mM	Standard	84 ± 0.989	81.5 ± 0.141^b^ ^,^ ^c^	30.75 ± 0.778	30.7 ± 0.283	367 ± 12.73	377 ± 2.828
		Osmolarity-Adjusted	85.8 ± 0.424	83.1 ± 1.55	31.0 ± 0.00	31.85 ± 0.778^c^	361 ± 1.141	383 ± 2.828
	55 mM	Standard	84.05 ± 0.353	82.05 ± 0.636^b^	31 ± 0.566	31.2 ± 0.141	369 ± 8.485	380.5 ± 4.949
		Osmolarity-Adjusted	85.6 ± 0.283	83 ± 0.848^b^	31.05 ± 0.071	30.85 ± 0.071	362.5 ± 2.121	371 ± 4.243
	110 mM		82.5 ± 0.00	81.55 ± 0.354^b^ ^,^ ^c^	30.8 ± 0.141	30.9 ± 0.141	373 ± 1.141	379 ± 0.00^c^
	165 mM		84.1 ± 0.566	81.3 ± 0.283^b^ ^,^ ^c^	30.7 ± 0.141	30.8 ± 0.141	365 ± 4.242	379.5 ± 2.12
PEG400	27.5 mM	Standard	86.00 ± 0.424^b^	83.45 ± 0.071^c^	29.05 ± 3.18	30.45 ± 0.707	337.5 ± 38.89	364.5 ± 0.707
		Osmolarity-Adjusted	87.55 ± 0.071^b^ ^,^ ^c^	85.6 ± 0.141^c^	31.2 ± 0.00	30.75 ± 0.071	356 ± 0.00	359 ± 0.000
	55 mM	Standard	87.2 ± 0.849^b^ ^,^ ^c^	85.15 ± 0.919^c^	30.7 ± 0.283	30.6 ± 0.00	351.5 ± 0.707	359.5 ± 4.95
		Osmolarity-Adjusted	88.15 ± 0.353^b^ ^,^ ^c^	85.8 ± 0.141^c^	30.9 ± 0.141	30.8 ± 0.141	351 ± 2.828	359 ± 2.828
	110 mM		89.6 ± 0.141^b^ ^,^ ^c^	86.35 ± 0.919^c^	30.6 ± 0.283	30.5 ± 0.280	341.5 ± 2.12	353.5 ± 6.36
	165 mM		88.6 ± 0.424^b^ ^,^ ^c^	84.00 ± 0.989	30.7 ± 0.141	30.25 ± 0.071	346.5 ± 3.54	361.0 ± 2.83

^a^ Data is reported as mean ± 1SD.

^b^ Significant difference relative to the unsupplemented (i.e., 0 mM) condition at the opposing temperature.

^c^ Significant difference relative to the unsupplemented (i.e., 0 mM) condition at the equivalent temperature.

Trehalose afforded no protection based on any of the assessed metrics; rather, it negatively impacted storage outcomes at both temperatures (Figures 2C,D). At −4°C, concentrations of 55 mM and above proved to significantly increase hemolysis relative to the unsupplemented control (Figure 2C). However, all concentrations led to a significant decrease in EI_max_ (Figure 2D) and MCV (Table 1). While trehalose did not increase hemolysis at 4°C, a similar concentration-dependent decrease in EI_max_ was seen (Figures 2C,D). Although, in contrast to the results at −4°C, no changes in MCV were evident at 4°C (Table 1). Similar to PEG400, no increase in hemolysis was apparent following the addition of trehalose, and thus it is assumed that these changes are storage-induced (Supplementary Table S1).

**FIGURE 3:**
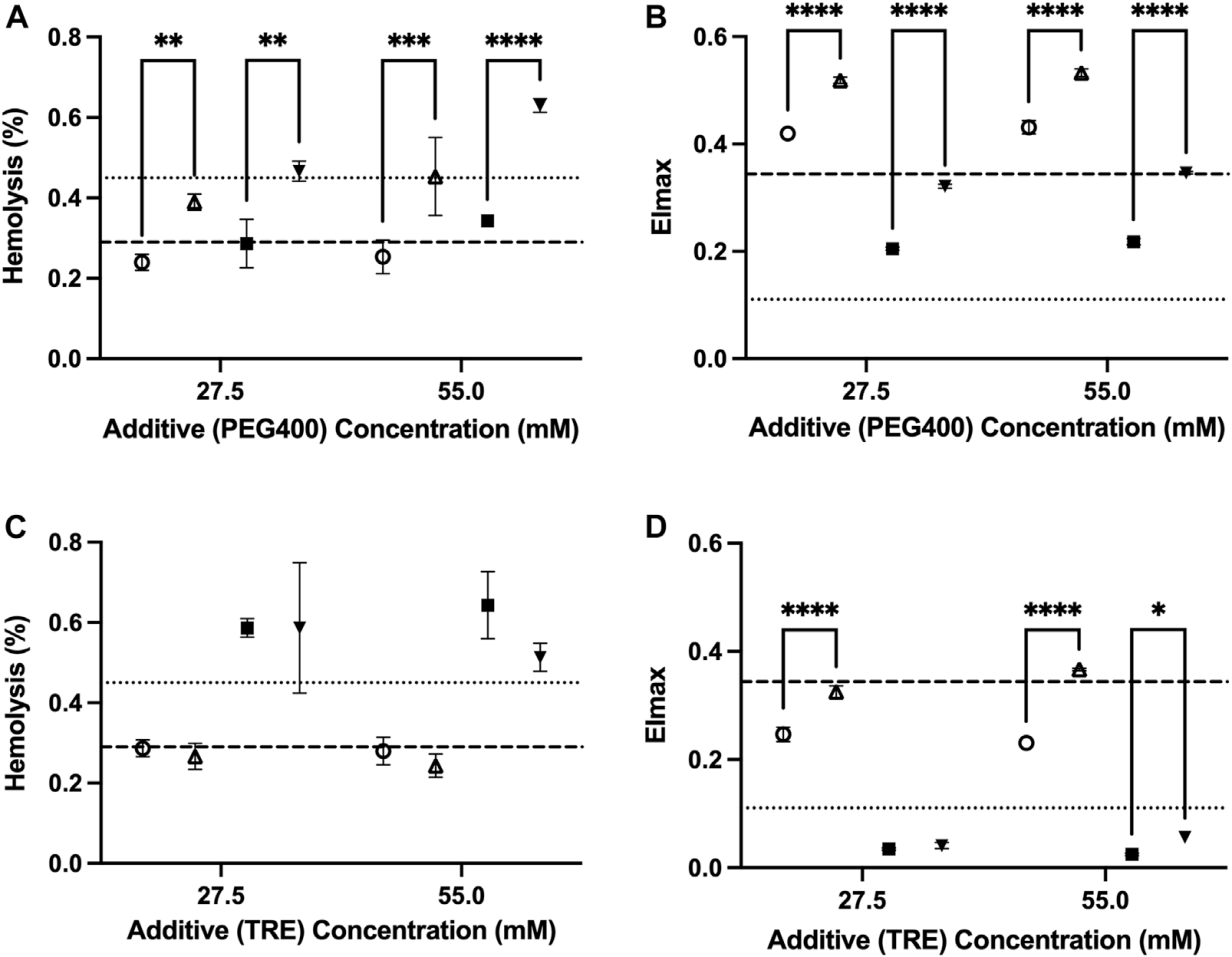
The role of extracellular osmolarity adjustments on the hemolysis and deformability of RBCs stored in PAG3M supplemented with either PEG400 or trehalose for 42 days at 4°C (○:standard; △:osmolarity-adjusted) and −4°C (■: standard; ▼:osmolarity-adjusted). **(A)** Hemolysis of PEG400-stored RBCs. **(B)** EI_max_ of PEG400-stored RBCs. **(C)** Hemolysis of trehalose-stored RBCs. **(D)** EI_max_ of trehalose-stored RBCs. Dashed lines represent the reference values from the un-supplemented (*i.e.*, 0 mM) conditions, with the heavy dashed line representing values at 4°C and the light dashed line representing values at −4°C. “4°C-adj.” and “-4°C-adj.” indicate the samples for which the osmolarity was adjusted through the removal of either 27.5 mM or 55 mM mannitol. In each case, the amount of mannitol removed was directly related to the concentration of the additive present such that the final osmolarity of the solution was unchanged. All error bars represent the standard deviation of three samples per condition. Error bars are not present in cases where they are smaller than the size of the symbol. Significant differences were calculated using a two-way analysis of variance (ANOVA) followed by a Sidak’s *post hoc* test: *p < 0.05; **p < 0.01; ***p < 0.001; ****p < 0.0001.

The osmolarity adjustments indicate that a portion of the damage from trehalose is osmolarity-dependent. The adjustment made to retain the osmolarity of the PAG3M solution in the presence of 55 mM trehalose (through not including 55 mM mannitol in PAG3M) caused a significant improvement in deformability at both temperatures (4°C: *p* < 0.0001; −4°C: *p* = 0.0108) (Figure 3D). While the osmolarity adjustment for the 27.5 mM trehalose-supplemented condition similarly improved deformability outcomes at 4°C (*p* < 0.0001), this was not the case at −4°C (*p* = 0.938) (Figure 3D). Nevertheless, these slight, albeit significant increases in EI_max_ did not correspond to reduced hemolysis in the case of trehalose (Figure 3D). For PEG400, osmolarity adjustments at both 27.5 mM and 55 mM caused a significant increase in hemolysis at both 4°C (27.5 mM: *p* = 0.00057; 55 mM: *p* = 0.0004) and −4°C (27.5 mM: *p* = 0.0011; 55 mM: *p* < 0.0001) (Figure 3A). Interestingly, this contrasted with the deformability outcomes which were improved in each case (*p* < 0.0001 for 27.5 mM and 55 mM) (Figure 3B). For both trehalose and PEG400 cases, the osmolarity adjustments did not promote any significant differences in the RBC hematological indices (Table 1).

## Discussion

It is expected that the diminished rate of biochemical reactions, and thus metabolic deterioration, should improve the quality of RBCs stored at supercooled temperatures (<0°C). Herein, we confirmed that loss of ATP, which is largely responsible for the hypothermic storage lesion at 4°C, is alleviated at −4°C over the course of 126 days. While it is intuitive that ATP utilization would be diminished at lower temperatures; interestingly, there was an initial rise in ATP levels at −4°C (as seen at the Day-21 timepoint-Figure 1A) that was absent at 4°C. It is well known that during the first 2 weeks of hypothermic storage, the high rate of 2,3-DPG breakdown results in increased 3-phosphoglycerate levels which subsequently drives late-stage glycolytic ATP production by 3-phosphoglycerate kinase. As the rate at which this occurs exceeds the rate of ATP utilization, an initial burst in ATP levels is expected. At −4°C, diminished 2,3-DPG breakdown combined with diminished ATP utilization could explain the rise in ATP seen at Day 21 and the lack of an equivalent trend at 4°C (as this burst likely occurred earlier in the storage period, between 7 and 21 days). Despite this, there was an observable degradation of many RBC quality parameters in RBCs stored at −4°C relative to those stored at 4°C. We therefore posit that this heightened damage at −4°C is not a result of the exacerbation of mechanisms responsible for the hypothermic storage lesion (of which many of the guiding forces are ATP-dependent). Rather, there must be alternate mechanisms of injury which materialize and predominate at −4°C. We, to an extent, reveal what these potential injury mechanisms could be based on some of the changes observed in the *in vitro* RBC quality parameters, or the lack thereof.

One of the notable differences between supercooled and hypothermic-stored RBCs was the extent to which deformability (EI_max_) was reduced during supercooling (Figure 1B). We suspect that the combined increase in ROS accumulation (due to the lower activation energy of reactions that generate free radicals vs. those that scavenge free radicals) as well as changes in the membrane phase behaviour (manifesting in more irreversible phospholipid aggregation upon rewarming), together, form the basis for this loss in deformability at −4°C (Suzuki and Mittler, 2006). During hypothermic storage, most ROS-damaged proteins/lipids are released as microvesicles (MVs) and while MV release was not evaluated as part of the present study, if indeed MV release were exacerbated at −4°C, this would likely manifest in a reduction in the MCH considering MVs are dense in hemoglobin (∼31.6 μM–69.3 μM; globin peptides are 30%–50% of all peptides in the sample) (Stapley et al., 2012; Risbano et al., 2015). Provided that no such differences in MCH were seen between the supercooled and hypothermic-stored RBCs, it is possible an increase in ROS damage was not associated with increased MV release (Figure 1D). Given the known inverse relationship that exists between temperature and the aggregation of liquid-ordered (Lo) domains which MVs are comprised of, the assumption that MV formation would be impeded at −4°C (relative to 4°C) is not far-fetched (Quinn, 1985; Pollet et al., 2018). Increased Lo domain aggregation at low temperatures would cause a corresponding rise in the Lo/Ld (*i.e.*, liquid-ordered/liquid-disordered) boundary line tension which would in turn make it energetically unfavourable for MV release to occur (Vind-Kezunovic et al., 2008). The possibility of diminished MV release and increased ROS accumulation at supercooled temperatures could therefore act in synergy to promote continuous accumulation of peroxidised lipids, and thereby decrease deformability. While further studies are warranted to confirm these hypotheses (*i.e.*, increased ROS formation, increased lipid peroxidation, and diminished MV release at low temperatures), they did serve to inform our choice of trehalose and PEG400 as additives to curb injury at −4°C.

PEG has proven to be an invaluable protective agent in a variety of different cell, tissue, and organ-based supercooling applications due to an uncharacteristic ability to minimize lipid peroxidation through either direct scavenging of peroxide radicals and/or shielding of membrane lipids from peroxidation (the precise mechanism still remains unclear) (Mack et al., 1991; Berendsen et al., 2014; Puts et al., 2015; de Vries et al., 2019). While PEG35 (*i.e.*, the molecular weight of 35,000 g/mol) is the most used subtype in many of these applications, data from one of our pilot studies indicated that PEG400 is preferable for the supercooled preservation of RBCs (Supplementary Figure S1). In fact, Zhu et al. (2022), in one of the few published studies describing supercooled RBC preservation, showed that PEG400 (75 mM) can diminish hemolysis during long-term storage at −8°C in the presence of 7.5%–10% (w/v) of glycerol (Zhu et al., 2022). Our study provides further evidence of the protection imparted by PEG400 in supercooled RBCs, with all tested concentrations (27.5–165 mM) providing an improvement in deformability with an increase in concentration at −4°C (Figure 2B). The pronounced increase in deformability in the 110 mM-PEG400-supplemented condition appeared particularly prevalent, even significantly increasing deformability relative to the un-supplemented condition at 4°C (Figure 2B). Interestingly, this was the only tested concentration that also diminished hemolysis at −4°C (unsupplemented: 0.45% ± 0.026%; 110 mM PEG400: 0.34% ± 0.02%) (Figure 2A). Despite enhancing RBC deformability, PEG400 osmolarity adjustments with mannitol led to increased hemolysis at both 4°C and −4°C (Figures 3A,B). This is not unexpected as mannitol could have direct antioxidant scavenging activities and osmoregulatory activities that PEG400 would lack (Beutler and Kuhl, 1988; Du et al., 2001). The latter is evidenced by the known fact that low-molecular weight forms of PEG can easily partition into the liquid-ordered (Lo) domain of the lipid, diminishing their capacity to provide osmoregulation. The possibility that PEG400 could be partitioning and increasing spacing in the Lo domain of the RBCs could explain the large rise in MCV when PEG400 is added to the RBCs and not washed out (Supplementary Figure S2A) (Momin et al., 2015). Thus, the increase in MCV seen at both temperatures could simply be an artifact of leftover membrane bound PEG400 enhancing the spreading of lipids in the membrane (Table 1); however, there does appear to be a return to a baseline MCV following the three wash steps that were performed prior to the assessment of hematological indices (Supplementary Figure S2A).

In contrast to PEG400, trehalose offered no protection at −4°C and appeared to increase hemolysis while promoting a loss in MCV and deformability in a concentration-dependent manner (Figures 2C,D). Surprisingly, this was less so the case at 4°C and was further alleviated by osmolarity adjustments with mannitol. Thus, the damage is not a result of biochemical toxicity and instead appears to be osmolarity-based. Adding to this point is the implausibility that a significant amount of trehalose would have permeated the RBC (the passive uptake of trehalose into RBCs only readily occurs above 34°C (Satpathy et al., 2004)) and the lack of evidence to suggest that trehalose partitions into lipid bilayers to the extent that PEG400 does (Villarreal et al., 2004). Some permeation and/or membrane partitioning may have occurred as seen by the increased MCV following trehalose addition (Supplementary Figure S2B), but this rise in MCV was nowhere near that which was seen when using PEG400 (Supplementary Figure S2A). However, it must be established whether the relative inability of trehalose to partition into the membrane/permeate the RBC limited its abilities to protect against the injury at −4°C, or whether its resulting functionality as an impermeant osmotic agent (relative to PEG400) impeded any protection it could impart. With respect to the latter, an increase in RBC shrinkage could promote activation of non-selective, volume-dependent cation channels, leading to high intracellular Cl-concentrations, which would cause intracellular acidification and a further decrease in the activation energy required for the reduction of major RBC antioxidant systems (Adediran, 1983; Huber et al., 2001). On the other hand, partitioning of the Lo phase could minimize low temperature-induced aggregation of Lo domains (and the damage that would result from this), while permeation of PEG400 could limit irreversible denaturation of proteins/macromolecules known to be prominent during prolonged supercooled storage through the formation of hydration shells (Privalov, 1990). Ultimately it is perhaps a combination of functionality as an impermeant osmotic agent, membrane partitioning, and/or cell permeation that together guide the protection afforded by PEG400 and the damage afforded by trehalose during supercooled storage.

The somewhat speculative nature of the preceding discussion highlights the major limitations of the present study. It is challenging to fully contextualize the results without molecular dynamics simulations to evaluate the interactions of PEG400/trehalose with the RBC membrane as well assessments of the RBC lipidome and metabolome (both with and without trehalose/PEG400) at each of the tested temperatures. Additional *in vitro* assessments of MV formation, morphology, intracellular ion concentrations, ROS, and intracellular pH are also warranted. Furthermore, of the available RBC storage solutions, it is possible that PAG3M is not the most optimal. PAG3M is designed in large part to minimize ATP depletion and intracellular acidification during hypothermic storage, but clearly this is less of a concern when RBCs are supercooled. How PAG3M ameliorates the supercooled storage lesion and whether design considerations for this storage solution could in any way exacerbate damage, is a necessary subject of future investigation. Again, this would warrant that some of the aforementioned evaluations be performed and that they even be done using some of the other available storage solutions.

Despite these limitations, we are investigating a novel paradigm of RBC storage, and therefore the fact that: i) ATP levels are better maintained, ii) non-metabolic sources of injury are augmented, and iii) injuries can be ameliorated by additives, are valuable outcomes when considered in combination. Together, they not only grant validity to supercooling as an RBC preservation strategy, but also highlight a dichotomy in the supercooled *versus* hypothermic storage lesions which warrant further mechanistic insight to the use of other types of additives (*e.g.*, antioxidants, metabolic inhibitors, cytoskeletal stabilizers), or even a redesign of the storage solution. When such measures are taken in our follow-up studies, the diminished progression of metabolism at high sub-zero temperatures presents an opportunity to improve the quality of stored RBCs and to preserve them for an extended period beyond 42 days.

## Data Availability

The original contributions presented in the study are included in the article/Supplementary Material, further inquiries can be directed to the corresponding authors.
