# Correction: Comparing two extracellular additives to facilitate extended storage of red blood cells in a supercooled state

**DOI:** 10.3389/fphys.2025.1717907

**Published:** 2025-10-16

**Authors:** Nishaka William, Ziya Isiksacan, Olga Mykhailova, Carly Olafson, Martin L. Yarmush, O. Berk Usta, Jason P. Acker

**Affiliations:** ^1^ Department of Laboratory Medicine and Pathology, University of Alberta, Edmonton, AB, Canada; ^2^ Center for Engineering in Medicine and Surgery, Massachusetts General Hospital, Harvard Medical School, Boston, MA, United States; ^3^ Shriners Children’s, Boston, MA, United States; ^4^ Innovation and Portfolio Management, Canadian Blood Services, Edmonton, AB, Canada; ^5^ Department of Biomedical Engineering, Rutgers University, Piscataway, NJ, United States

**Keywords:** supercooling, storage lesion, red blood cells, trehalose, polyethylene glycol, surface-sealing, nucleation

Centrifugation speeds that should be noted as “1000 g, acc. 3, **dec. 9**” instead appear as “1000 g, acc. 3, **December 9**”. A correction needs to be made to the section titled “Blood processing and preparation for supercooled storage” as well as “RBC *in vitro* quality assays”. Both are subsections within the methods section.

The original article has been updated.

