# Supplementary material for: Comparing two extracellular additives to facilitate extended storage of red blood cells in a supercooled state
Source: Front Physiol. 2023 Jun 1;14:1165330. doi: 10.3389/fphys.2023.1165330 (PMC10267403; doi:10.3389/fphys.2023.1165330)
Supplement: Supplementary file 1 [file DataSheet1.docx]

|  | | | Hemolysis (%) |
| --- | --- | --- | --- |
| No Additive | | | 0.11 ± 0.006 |
| Trehalose | 27.5 mM | Standard | 0.11 ± 0.023 |
|  |  | Osmolarity-Adjusted | 0.16 ± 0.012 |
|  | 55 mM | Standard | 0.14 ± 0.035 |
|  |  | Osmolarity-Adjusted | 0.16 ± 0.015 |
|  | 110 mM | | 0.15 ± 0.072 |
|  | 165 mM | | 0.18 ± 0.021 |
| PEG400 | 27.5 mM | Standard | 0.19 ± 0.081 |
|  |  | Osmolarity-Adjusted | 0.19 ± 0.010 |
|  | 55 mM | Standard | 0.18 ± 0.010 |
|  |  | Osmolarity-Adjusted | 0.23 ± 0.026 |
|  | 110 mM | | 0.14 ± 0.006 |
|  | 165 mM | | 0.15 ± 0.031 |

Table S1. Day 0 Hemolysis

Figure S1. The impact of commonly used PEG subtypes on the (A) hemolysis and (B) deformability of PAG3M-stored RBCs following 21 days of storage at -4 °C. PEG concentration was 5% (w/v) in each condition. Hemolysis on Day 0 was as follows for each different subtype: 400 g/mol – 0.147 ± 0.031; 8000 g/mol – 0.147 ± 0.015; 0.173 ± 0.006. Dashed line indicates the respective values from the unsupplemented (ie. 0 mM PEG) condition. All error bars represent the standard deviation of three samples per condition and significant differences were calculated using a one-way analysis of variance (ANOVA) followed by a Tukey’s post-hoc test: **p* < 0.05; ***p* < 0.01.

Figure S2. Changes in MCV following the addition and removal of (A) PEG400 and (B) trehalose. Three wash steps (1000 g, 9 acc., 3 dec.) were performed for both the addition (ie. unwashed) and removal (ie. washed) of the compounds. For compound addition, all wash steps consisted of the PAG3M supplemented with the solutions of interest, whereas wash steps for the removal were all performed in the base-PAG3M without any PEG400 or trehalose. All error bars represent the standard deviation of three samples per condition and significant differences were calculated using a one-way analysis of variance (ANOVA) followed by a Tukey’s post-hoc test: **p* < 0.05; ***p* < 0.01; ****p* < 0.001; *****p* < 0.0001.
